# Special Issue “Absorbable Metals for Biomedical Applications”

**DOI:** 10.3390/ma14143835

**Published:** 2021-07-09

**Authors:** Hendra Hermawan, Mehdi Razavi

**Affiliations:** 1 Department of Mining, Metallurgical and Materials Engineering, Université Laval, Quebec City, QC G1V 0A6, Canada; 2 Biionix^TM^ (Bionic Materials, Implants & Interfaces) Cluster, Department of Internal Medicine, College of Medicine, University of Central Florida, Orlando, FL 32827, USA; Mehdi.Razavi@ucf.edu

Current temporary metal implants made from titanium or stainless steel are not absorbable. Thus, a second surgery is needed to remove the implants after the tissue heals, or (for children) after they outgrow their implants. This second surgery is costly and involves additional risks of infections and pain for the patient. Therefore, absorbable metals are currently generally preferred and being investigated. Absorbable metals have shown significant clinical potential as an alternative to polymers in implant applications, where the material is eventually replaced by healthy, functioning tissue. However, several challenges remain before these metals can be translated to humans. First, the metal alloys with sufficient strength contain aluminum, yttrium, or lithium, all of which pose serious concerns for long-term toxicity. Second, in some cases such as magnesium alloys, they degrade too rapidly, and as a result, also generate possibly harmful hydrogen gas pockets. Consequently, these implants lose their mechanical integrity before the host tissue heals.

Innovations and further improvements are required, especially for load-bearing implants. The main focus of this Special Issue is to therefore collect scientific contributions dealing with the development of absorbable metals with improved and unique corrosion and mechanical properties for applications in highly loaded implants, or cardiovascular and urethral stents. This Special Issue assembles a group of highly original manuscripts that present a range of exciting innovations in alloying [1,2,3] and compositing [4], along with their testing and assessments [5,6,7] to introduce novel medical implants based on magnesium [2,3,4,8,9], zinc [1,7], or iron [10,11]. As the biointerface plays an important role in implant–tissue interactions, contributions to implant coating and surface engineering strategies and their effects on the implant properties and corrosion are also discussed [8,10,12]. Two studies on mechanical testing of implants made of biodegradable polymer composites provide a complimentary benchmark toward clinical application of absorbable metal implants [13,14].

Finally, the concept of absorbable metal implants might result in a significant impact on the future work of standardization agencies. However, standardization must be balanced with the main challenge in the field, which remains the successful translation of this innovative concept into medical products that guarantee the growth of the absorbable metal field. Genetic-based methods and biomarkers should be studied with more depth to evaluate their biocompatibility and bioactivity. 

Contributions have been solicited from scientists working in the fields of biomaterials, tissue engineering, bioengineering, and medicine. Finally, the Editors give special thanks to the authors, and to the editorial team of *Materials*, for collaborative and peer-review process. 

We hope you will enjoy reading this issue as much as we had the pleasure of assembling it.
